# Plasmonic circular resonators for refractive index sensors and filters

**DOI:** 10.1186/s11671-015-0913-4

**Published:** 2015-05-08

**Authors:** Wei Wei, Xia Zhang, Xiaomin Ren

**Affiliations:** State Key Laboratory of Information Photonics and Optical Communications, Beijing University of Posts and Telecommunications, P. O. Box 66, Beijing, 100876 China

**Keywords:** Surface plasmons, Sensors, Filters

## Abstract

A plasmonic refractive index sensor based on a circular resonator is proposed. With all three dimensions below 1 μm, the sensor has a compact and simple structure granting it ease-of-fabrication and ease-of-use. It is capable of sensing trace amounts of liquid or gas samples. The sensing properties are investigated using finite elements method. The results demonstrate that the plasmonic sensor has a relatively high sensitivity of 1,010 nm/RIU, and the corresponding sensing resolution is 9.9 × 10^−5^ RIU. The sensor has a relatively high quality factor of 35, which is beneficial for identifying each transmission spectrum. More importantly, the sensitivity is not sensitive to changes of structure parameters, which means that the sensitivity of the sensor is immune to the fabrication deviation. In addition, with a transmittance of 5% at the resonant wavelength, this plasmonic structure can also be employed as a filter. In addition, by filling material like LiNbO_3_ or liquid crystal in the circular resonator, this filter can realize an adjustable wavelength-selective characteristic in a wide band.

## Background

At present, more and more researchers pay their attentions on the investigation of surface plasmons (SPs). Thus, a lot of articles about SPs emerged, from which numerous devices based on SPs are proposed. SPs are optically induced oscillations of the free electrons at the surface of a metal and can confine and propagate electromagnetic energy far beyond the diffraction limit for electromagnetic waves in dielectric media [1,2]. This could lead to miniaturized photonic components with dimensions scale much smaller than those currently achieved [3,4], such as plasmonic waveguides [5-8] and plasmonic nanolasers [9,10]. Due to the susceptibility of SPs to surrounding dielectric, SPs and surface plasmon resonance (SPR) exhibit excellent properties for sensing applications [11-13]. In past decades, various plasmonic sensors based on SPs and SPR have been proposed and investigated [14-24], especially prism-coupled SPR sensors [14-17] and fiber-coupled SPR sensors [20-24]. Generally, dimensions of prism-coupled SPR sensors are huge for integration of sensors on chips and their sensitivities are not high compared to fiber-coupled SPR sensor. Although fibers make SPR sensors smaller and more flexible, the dimensions are still too large for sensing applications of lab on chip. Recently, metal-insulator-metal (MIM) plasmonic waveguides offering very high optical confinement and closer spacing to adjacent waveguides or structures have been proposed for diverse applications, such as MIM optical filers [25,26], electromagnetically induced transparency [27,28], Bragg gratings [29,30], and directional couplers [31,32]. Compared to other sensors, plasmonic sensors with MIM structures have an inherent advantage to achieve high integration.

In this paper, we focused on the compactness of the sensor with an acceptable sensitivity and its integration to other components. By employing the MIM structure, we proposed a plasmonic refractive index sensor based on a circular resonator. This sensor has a simple and ultra-compact structure. It is comprised of a circular resonator and a bus waveguide. All three dimensions of the sensor are below 1 μm. Combing such a compact structure and sensing capability of SPs, this plasmonic refractive index sensor can realize real-time and on-chip sensing. Moreover, the structure parameters have neglected impact on sensing sensitivity of the plasmonic sensor. Besides the application of sensors, this structure can be used as filters due to its low transmittance at the resonant wavelength. Moreover, by filling material like LiNbO_3_ or liquid crystal, the filter can realize an adjustable wavelength-selective characteristic in a wide band.

## Methods

**Figure 1:**
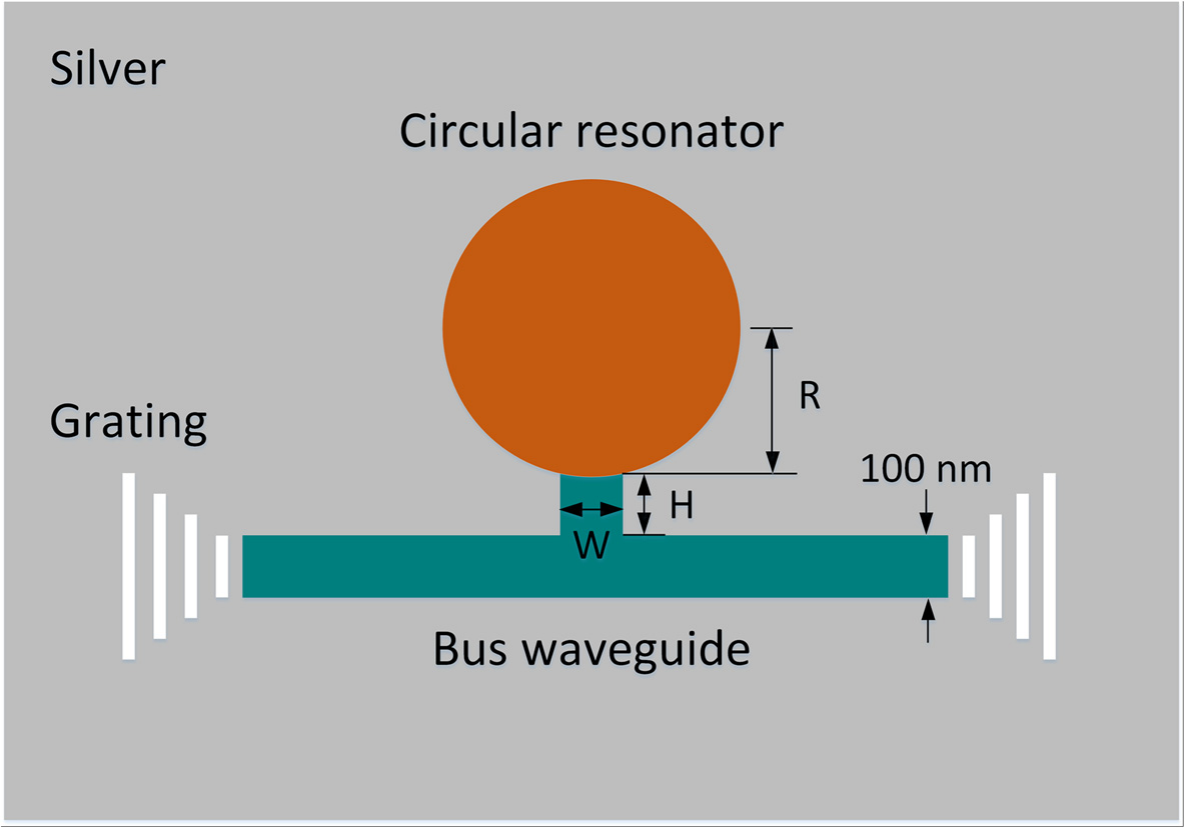
Schematic diagram of the plasmonic structure.

The schematic of the plasmonic structure is demonstrated in Figure 1, where the background material in gray is silver, whose permittivity is described by the Drude model ${\varepsilon}_{\mathrm{r}}={\varepsilon}_{\infty }-{\omega}_{\mathrm{p}}^2/\left({\omega}^2+j\gamma \omega \right)$ , with *ε*_∞_ = 3.7, *ω*p = 9.1 eV, and γ = 0.018 eV [33]. The parameters adopted here fit the experimental data at the infrared frequencies [34]. The long strip waveguide filled with silica in the silver pad is called bus waveguide. The circle above the bus waveguide is a circular resonator behaving as a Fabry-Perot (F-P) cavity. The empty circular resonator in the silver pad is used to collect the analyte (liquid or gas) to be sensed. When this structure is used as a filter, the empty circular resonator can be filled by liquid with proper refractive index, LiNbO_3_ or liquid crystal, to realize customized or adjustable wavelength-selective characteristic. The small gap between the bus waveguide and the circular resonator is designed to enhance coupling between them. The width of the bus waveguide is 100 nm. In spite of transmission loss in the waveguide, the length of the bus waveguide has no influence on sensing and wavelength-selective characteristics of this plasmonic structure, so its length is fixed at 800 nm considering the compactness and integration. The radius of the circular resonator is *R*, and the height and width of the small gap are denoted by *H* and *W*, respectively. The gratings at the start and end of the bus waveguide are used to couple super-continuum light into the sensor and the transmission spectrum out of the bus waveguide. This plasmonic structure could be fabricated by steps as follows: first, deposit an Ag film with a thickness of 500 nm on a silica substrate; then, fabricate the required pattern by EBL and etching, and deposit a silica film with a thickness of 500 nm; last, clear the redundant silica on the Ag film and in the circular resonator, and fabricate the gratings at the two ends of the bus waveguide by EBL and etching.

## Results and discussion

In this section, we first investigated the sensing properties of the plasmonic structure and then indicated its wavelength-selective properties as a filter at the end of this section. Sensing properties including distribution of electromagnetic field, transmission spectrum, and sensitivity are numerically analyzed using finite elements method with scatter boundary conditions. In the calculation, a plane wave was injected from the left side of the bus waveguide by a port to excite fundamental TM modes of SPs. The transmitted light was collected from the right side of the bus waveguide which is defined as T = P_out_/P_in_, where Pin = ∫*PoavzdS*_1_ and Pout = ∫*PoavzdS*_2_; *Poavzd* is the z component of time-average power flow. The transmission spectra of the plasmonic structure are obtained by parametrically sweeping the input wavelength with step of 1 nm.

**Figure 2:**
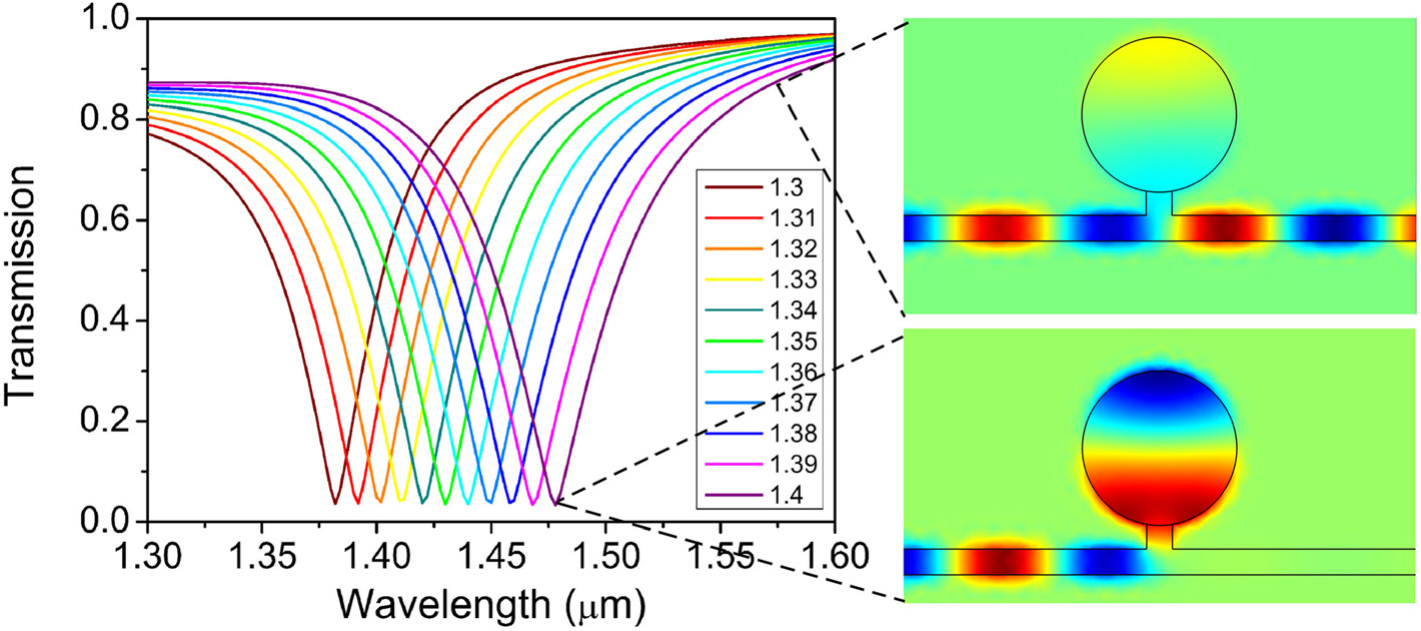
Transmission spectra of the plasmonic refractive index sensor for varying refractive index of analyte. Insets are magnetic field *H*_z_ corresponding to the index of 1.4 at the wavelengths of 1.476 and 1.576 μm.

For various refractive indices of analyte in the circular resonator, Figure 2 demonstrates the corresponding transmission spectra of this plasmonic sensor with a structure of *R* = 300 nm, *H* = 100 nm, and *W* = 100 nm. For the transmission spectrum corresponding to the refractive index 1.4, there is a dip in the transmission spectrum and most other wavelengths of the injected wideband light are transmitted through the bus waveguide. The wavelength corresponding to the deepest dip is called the resonant wavelength. At the resonant wavelength, most energy coupled into the circular resonator from the bus waveguide. As the insets show in Figure 2, at the wavelength of 1.576 μm, most energy transmitted through the bus waveguide without coupling into the circular resonator. But at the wavelength of 1.476 μm, most energy coupled into the circular resonator and very little energy transmitted through the bus waveguide. The energy resonated in the circular resonator is sensitive to the refractive index variations of the analyte in the circular resonator, so when the refractive index of the analyte was changed, the resonant wavelength in the transmission spectrum shifted which is demonstrated in Figure 2. Thus, the above is the sensing mechanism of this plasmonic refractive index sensor.

**Figure 3:**
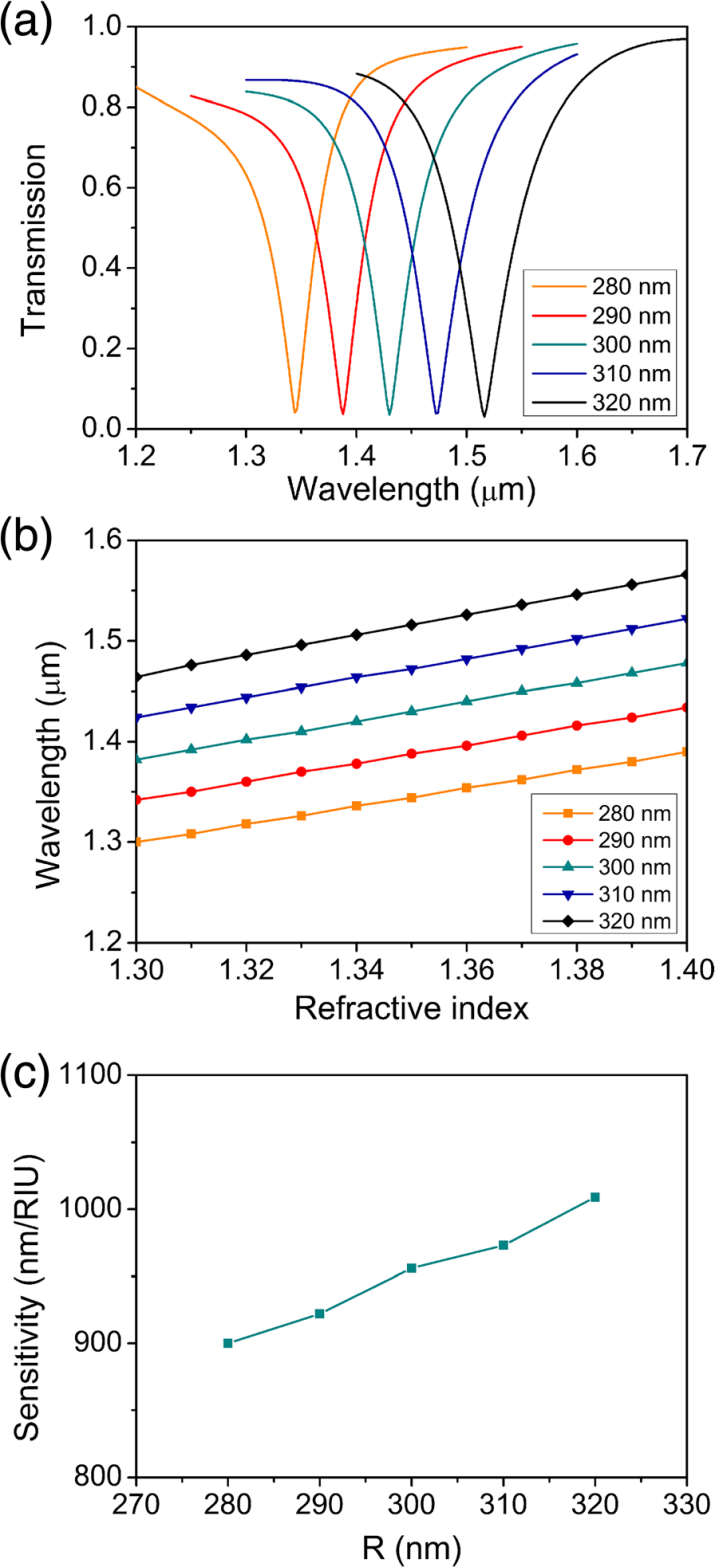
Sensing properties as functions of *R*. **(a)** Transmission spectra of refractive index 1.35 as functions of *R*. **(b)** Shifting wavelengths corresponding to the dips in the transmission spectra for different *R*. **(c)** Sensitivities of the plasmonic refractive index sensors for different *R*.

Transmission spectra of refractive index 1.35 for values of *R* varying from 280 to 320 nm are demonstrated in Figure 3a. The transmission dip shifts towards a longer wavelength with incremental *R*. The wavelength difference between each resonant wavelength is about 35 nm. For each transmission spectrum, the shift of the resonant wavelength can be explained via the standing-wave condition, *Nλ*N = 2*n*eff*L*, *N* = (1, 2, 3 …). For a specific *N*, larger radius of the circular resonator causes the red shift of resonant wavelength, while shorter radius of the circular resonator causes the blue shift of resonant wavelength. The resonant wavelength versus refractive index of analyte for different *R* values is demonstrated in Figure 3b. All the resonant wavelengths increase linearly as the refractive index increases, and slopes of the five lines are similar. The sensitivity of the plasmonic sensor was calculated using *S*λ(nm/RIU) = |*dλ*peak/*dn*a|, and shown in Figure 3c. The sensitivity of the plasmonic refractive index sensor has a positive correlation with incremental *R* in a range from 900 to 1,010 nm/RIU. The maximum sensitivity is 1,010 nm/RIU and its corresponding sensing resolution is 9.9 × 10^−5^ RIU. Thus, the radius of the circular resonator influences not only the resonant wavelength but also the sensitivity. However, the influence of the radius of the circular resonator on the sensitivity of the plasmonic refractive index sensor is very small and can be neglected, the variation of sensitivity is in a range of 110 nm/RIU around 950 nm/RIU (fluctuation is about 6%).

**Figure 4:**
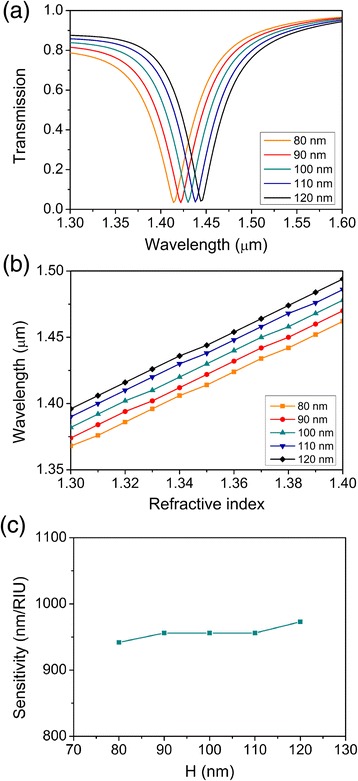
Sensing properties as functions of *H*. **(a)** Transmission spectra of index 1.35 for *H* varying from 80 to 120 nm. **(b)** Shifting resonant wavelengths for *H* varying from 80 to 120 nm. **(c)** Sensitivities of the plasmonic sensors for *H* varying from 80 to 120 nm.

Next, we analyzed the impact of different *H* on sensing properties of the plasmonic refractive index sensor. Transmission spectra of index 1.35 for values of *H* varying from 80 to 120 nm are demonstrated in Figure 4a. With incremental *H*, the resonant wavelength shifts towards a longer wavelength in a small range from 1.414 to 1.444 μm. The wavelength difference between each resonant wavelength is about 6 nm. This shift can be explained by associating with the above standing-wave condition of the F-P resonator. The incremental *H* actually increases the length of the F-P resonator, leading to the shift of the resonant wavelength towards a longer wavelength. The resonant wavelength versus the refractive index of analyte for different *H* values is demonstrated in Figure 4b. All the resonant wavelengths increase linearly as the refractive index of analyte increases, and the slopes of the five lines are similar. The sensitivity of the plasmonic sensor as a function of *H* is shown in Figure 4c. With incremental *H*, the sensitivity of the plasmonic refractive index sensor gradually increases in a small range from 942 to 973 nm/RIU around 960 nm/RIU (fluctuation is about 1.8%). Thus, the height of gap *H* influences only the resonant wavelength. The little influence of the height of the gap between the circular resonator and the bus waveguide on the sensitivity of the plasmonic refractive index sensor can be neglected.

**Figure 5:**
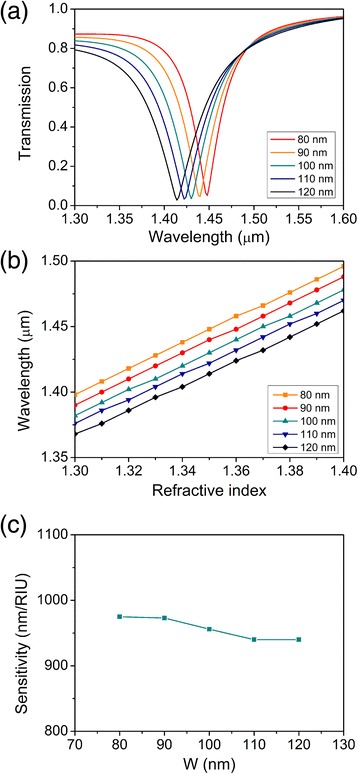
Sensing properties as functions of *W*. **(a)** Transmission spectra of index 1.35 for *W* varying from 80 to 120 nm. **(b)** Shifting resonant wavelengths for *W* varying from 80 to 120 nm. **(c)** Sensitivities of the plasmonic sensors for *W* varying from 80 to 120 nm.

The impact of different *W* on sensing properties of the plasmonic refractive index sensor was investigated, and transmission spectra of index 1.35 for values of *W* varying from 80 to 120 nm are demonstrated in Figure 5a. With the incremental *W*, the transmission dip shifts towards a shorter wavelength in a small range from 1.414 to 1.448 μm, which is contrary to the relation between transmission dip shift and *H*, and the wavelength difference between each resonant wavelength is about 7 nm. This shift can also be explained by associating with the above standing-wave condition of the F-P resonator. Widening *W* equivalently decreases the length of the F-P resonator, leading to the shift of the resonant wavelength towards a shorter wavelength. The resonant wavelength versus the refractive index of analyte for different *W* values is demonstrated in Figure 5b. All the resonant wavelengths increase linearly as the refractive index of analyte increases, and the slopes of the five lines are similar. The sensitivity of the plasmonic sensor as a function of *W* is shown in Figure 5c. With incremental *W*, the sensitivity of the plasmonic refractive index sensor gradually decreases in a small range from 940 to 975 nm/RIU around 960 nm/RIU (fluctuation is about 2%). Thus, the height of gap *W* influences only the resonant wavelength. The little influence of the width of the gap between the circular resonator and the bus waveguide on the sensitivity of the plasmonic refractive index sensor can be neglected.

At last, we will discuss the wavelength-selective characteristic of this plasmonic structure and indicate its application as a filter. As shown in Figure 2, the transmittance at the dip of the transmission spectrum is 5% and the central stop-wavelength shifts towards a longer wavelength with incremental refractive index of analyte. But the transmittance at the central stop-wavelength keeps the same. By filling material like LiNbO_3_ or liquid crystal, the central stop-wavelength can be adjustable. As shown in Figures 3, 4, and 5, the central stop-wavelength shifts with different structure parameters and the transmittance at the central stop-wavelength nearly keeps unchanged. So, by designing proper structures, the wavelength-selective characteristic can be customized.

## Conclusions

We have proposed a plasmonic circular resonator for refractive index sensor and filter. With all three dimensions below 1 μm, it has a simple and ultra-compact structure, which makes it easy to be filled with analyte and integrate with other components. The sensing properties of the proposed sensor are numerically analyzed using finite elements method. The positions of transmission dips have linear relations with the refractive index of analyte. The maximum sensitivity is 1,010 nm/RIU, its corresponding sensing resolution is 9.9 × 10^−5^ RIU. It has a relatively high quality factor of 35. More importantly, the sensitivity of this ultra-compact plasmonic sensor is immune to the changes of structure parameters. At last, we indicated that with a very low transmittance of 5%, this plasmonic structure could be also employed as a filter. And the transmittance keeps nearly unchanged when the structure parameters vary. By filling material with proper refractive index, LiNbO_3_ or liquid crystal, into the circular resonator, this filter can realize customized and adjustable wavelength-selective characteristic.

## Abbreviations

SPs: surface plasmons
MIM: metal-insulator-metal
F-P: Fabry-Perot
FEM: finite elements method
